# Comparison of the Importance and Prioritization of Information Communicated to Consumers by Experts Regarding Food Safety

**DOI:** 10.14252/foodsafetyfscj.D-21-00010

**Published:** 2022-05-13

**Authors:** Itsuko Horiguchi, Kazuo Koyama, Azusa Hirakawa, Mieko Shiomi, Kaoruko Tachibana, Katsuyuki Watanabe

**Affiliations:** 1 The Support Center for Clinical Pharmacy Education and Research, Tokyo University of Science, 1-3 Kagurazaka, Shinjuku-ku, Tokyo 162-8601, Japan; 2 Food Safety Commission Secretariat, Cabinet Office, Government of Japan, Akasaka 5-2-20, Minato-ku, Tokyo 107-6122, Japan; 3 Department of Environmental Medicine, Kochi Medical School, Kohasu, Oko-cho, Nankoku-shi, Kochi783-8505, Japan

**Keywords:** food safety, Delphi method, health foods, food poisoning, risk communication

## Abstract

Key topics related to risk communication and food safety were investigated by three different expert groups. In this study, the Delphi method was used to systematically and iteratively aggregate experts’ opinions, and the topics to be communicated to consumers were expressed and prioritized. The opinions of three groups, consisting of 26 members of the expert committee (EC) from the Food Safety Commission of Japan (FSCJ), 29 local government officials (LGOs) from their respective food safety departments, and 25 food safety monitors (FSM) appointed by the FSCJ, were obtained in the period of June through September 2017. “Safety and security concept” was identified and ranked high in all groups. This topic identified “Zero-risk” demand of consumers without understanding risks as the reverse side of safety. The EC group prioritized additional issues, such as “concept of risk” and “safety costs and relevant risk management”. The LGO and FSM groups prioritized specific hazard items for food poisoning and preventive measures. With regard to the so-called “health foods”, the EC and LGO groups indicated insufficient transmission of scientific evidence from the government to consumers, and the FSM group indicated insufficient understanding by consumers of the food labeling system for health and nutrition. Because consumers do not fully understand all concepts of food safety, governments are encouraged to disseminate the probability of risk and the knowledge of risk reduction directly to the consumers by using simple and easy-to-understand terms.

## Introduction

Food Safety Commission of Japan (FSCJ) was established on July 2003 under the Cabinet Office, Government of Japan, as an independent organization from risk management body. The main mission of the FSCJ is to implement science-based risk assessments on foods. Another important mission is risk communication about risk assessment results and scientific findings on food safety. More specifically, the FSCJ disseminates basic knowledge on food safety and the assessment results by using various sources, including its website and leaflets. Activities such as seminars and symposiums for consumers, meetings with stakeholders, and workshops for school children have been conducted to meet its communication purposes. The FSCJ also offers continuous communication to further outer bodies via regular meetings with media groups, consumer groups, and local government officials (LGOs). It provides phone call reception service from general consumers for inquiry about food safety. The FSCJ-hosted workshops have been lately expanded to include dietitians who are working in schools. Its-hosted lectures have been given to the food industry. These risk communication activities are carried out in accordance with the report titled “How to implement risk communication of food safety”^1^^)^.

Essential parts of risk communication include providing responses to concerns and interests of consumers and other stakeholders. The FSCJ has been conducted annual web surveys of food safety monitors (FSMs) since 2004 to understand what consumer concerns are^2^^)^. Recent survey results revealed that food poisoning was the greatest concern, followed by health foods. Food poisoning has always been a key concern throughout the surveys.

Various surveys have been conducted in Japan to verify consumer knowledge of food safety^3^^,^^4^^)^. The survey by Nakagaki et al^3^^)^ targeted LGOs to understand their knowledge in food hygiene departments in 2007. Masuyama et al^4^^)^ has conducted a surveillance on FSCJ experts in 2009. In these surveys, communicated topics regarding to food safety were prioritized by using the Delphi method. In the former survey^3^^)^, the highest priority topic among consumers was “risk of raw food”, followed by “food poisoning” and “food labeling”. In the survey^4^^)^ on FSCJ experts, the highest priority was given to “concept of risk” regarding food safety, followed by “pesticide residues” and “genetically modified crops and foods”. The matters that need communication with consumers differed depending on their social fields.

Risk communication should take place widely with contribution of all food safety leaders, including those in government and local influencers. Thus, this survey was conducted to know what types of concerns are prominent among three distinct groups, namely, expert committees (EC) such as FSCJ’s EC, who carry out risk assessments, LGO as front-line authorities to contact with food-related businesses, and FSM as a part of consumers.

## Methods

### Participants

Participants in this survey consisted of three distinct groups: ECs from the FSCJ (EC group), LGOs from their food safety departments (LGO group), and FSMs nominated by the FSCJ (FSM group). Members of the EC group had original affiliation with universities and/or research institutes in Japan, while providing expertise for FSCJ’s risk assessments. Two to three members were randomly selected from 12 Expert Committees to take part in this survey. Members of the LGO group for this survey were chosen from 47 prefectures’ LGOs across the nation. They were often experienced with in-person contacts with consumers and food business personals. Members of the FSM group were selected from food company workers and registered dietitians by the annual public offering^5^^)^. Members of the LGO and FSM groups were selected randomly. During the selection, their numbers were adjusted in proportion to the regional population among 10 blocks of the country. Instruction manual and a letter of consent to the framework of this study were provided to all selected persons via e-mail. In total, 26 EC members, 29 LGOs, and 25 FSMs have consented to participation in this survey.

### Survey with the Delphi Method

**Fig.1.**
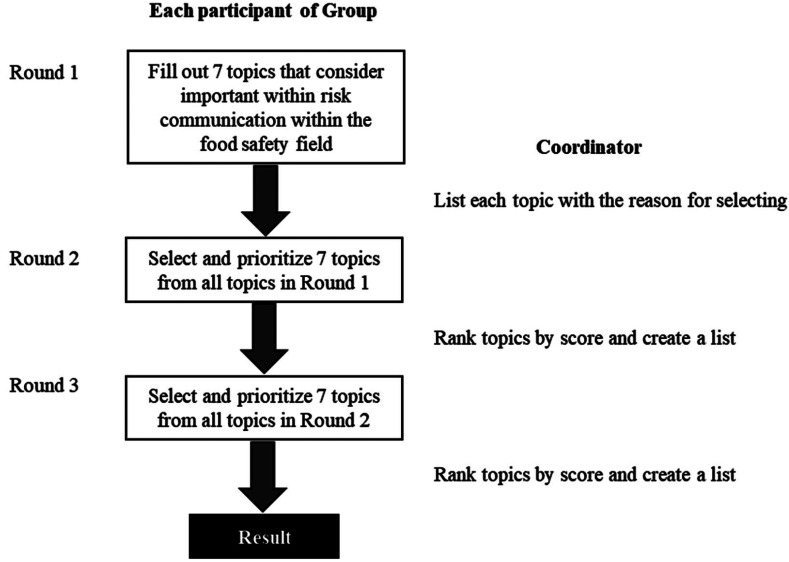
Flow chart of process for prioritizing concern topics in risk communication within the food safety field using the Delphi method. The score of each topic in subsequent Round is the total among 7 to 1 point assigned to 1st to 7th topics that participants ranked the seven topics in previous Round.

The survey analysis was conducted by using the Delphi method^6^^)^. A qualitative survey containing multiple questionnaires were given to each group. The steps in this survey are shown in Fig. 1. In Round 1, participants responded to seven topics that they considered important in risk communication in the food safety field. Fifteen days of respond period was allowed for Round 1 questionnaire (June 15 - 29, 2017), and the answers were submitted by e-mails. We then categorized and listed the topics by each group. The topics by the participants were maintained in the original text.

In Round 2, participants were asked to select seven top topics from all the choices listed in Round 1 questionnaire, and to rank them from the top 1 to 7 places. Participants in Round 2 were given 22 days to answer (August 24 - September 13, 2017). Remainder e-mails for respondents were sent three times, on September 1, 7, and 11, 2017. Then, the received answers were analyzed by weighed value average, by assigning 7 points to the 1^st^ ranking topic, while the lower-ranked topics were given gradually decreased point of integral number, to the point of 7^th^ ranking topic receiving 1 point. The scores of each topic present the sum of acquired points. Topics of concerns were listed in order from the highest to the lowest in this manner.

In Round 3, participants were asked to review the ranking of topics from the Round 2 and to re-rank from the first to the seventh places. Topics with no assigned points at Round 2 were excluded from the Round 3 list. Sixteen days of consideration period was allowed for Round 3 (September 26 - October 11, 2017). Reminding e-mails to urge submission were sent on October 3 and 10, 2017. All collected answers were used to gain weighed averages as described in Round 2. 

Public opinions were simultaneously gained for this survey, by using “Nopi”, a website tool appropriate for this purpose by the Cabinet office, Government of Japan. This tool enables researchers to collect public opinions while keeping anonymity of the responders. This study model was approved in 2019 by the Ethical Review Board of Life Science Promoting Association, a Public Interest Incorporated Foundation (Approval No. E2020-1).

## Results

The present study’s results consists of those from respondents including 26 participants of EC group, 29 participants of LGO group, and 25 participants of FSM group. The response rates of each round by group are shown in Table 1. They ranged from the lowest 79.3% to the highest 100%. The results of Rounds 1 and 2 are summarized in Tables 2a**–4b**. The right column of the tables shows the number of participants who selected the same topic in Round 1. The left column shows the rankings results of Round 2 from replies of participants who participated in all the topics of Round 1.

**Table 1.** The number of participants (the response rates, %) for each round and each group

Group	Expert Committee	Local Government Officials	Food Safety Monitors
Number of participants	26	29	25
Round 1	25 (96.2)	26 (89.7)	24 (96.0)
Round 2	26 (100.0)	23 (79.3)	22 (88.0)
Round 3	24 (92.3)	27 (93.1)	24 (96.0)

**Table 2:** a. Round 1 and Round 2 topics ranked by the EC group

Round 2^a)^			Round 1^a)^
Rank	Scores^b)^	Topics	Number of people on the same topic^c)^
1	70	Concept of risk	2
2	55	Health foods	6
3	53	The difference between safety and relief	1
4	31	Safety costs and relevant risk management	1
5	29	Food poisoning caused by natural toxins (animal- and plant-based)	3
6	27	Artificial products vs. natural products	2
7	23	Difference between acute and chronic effects	1
	23	Radioactive materials in foods	2
9	21	Food additives	3
10	19	Causes and prevention of food poisoning	1
	19	Eating of raw food (meat/fish)	2
	19	Genetically modified foods	2
	19	Food allergies	5
	19	Imported food safety	1
15	18	Food poisoning by microorganisms	4
16	17	Actual conditions and countermeasures of drug-resistant bacteria	1
17	16	Correct knowledge of food labeling	1
18	15	Excess of provisional standards for pesticides	1
	15	Prioritizing concept in food safety standards and risk communication	1
20	13	The safety of *gibier* as a food	2
	13	Risk of sweetened beverages	1
	13	Food contamination by post-harvest pesticides	1
23	12	Acrylamide in foods generated through heating	2
	12	Risk of natural products (hijiki arsenic, vanadium water, gluten, etc.)	1
25	10	Food poisoning by *Listeria monocytogenes*	2

^a)^ Number of participants in Round 1 and Round 2 were 25 and 26, respectively.

^b)^ There were 12 consenters on the first topic, and no consenters on the 57th topic.

^c)^ The total number of consenters for all topics was 100.

**Table 2:** b. (*Continued*)

Round 2			Round 1
Rank	Scores	Topics	Number of people on the same topic
26	9	Food poisoning by enterohaemorrhagic *E. coli*	2
	9	Eating of raw food (meat)	2
28	8	Food poisoning by *C. jejuni* and *C. coli*	4
	8	Food poisoning by *Norovirus*	3
30	7	Infant botulism	2
	7	Risk assessment for arsenic in foods	1
	7	Type A Trichothecene mycotoxin	1
	7	Mass media report	1
34	6	Food poisoning by natural toxins (plant-based)	7
	6	Food poisoning by *Clostridium botulinum*	1
	6	Food poisoning by *Eumycetes*	1
37	5	Substances contained in food that change to carcinogens after heating	1
	5	Quantity and quality of artificial synthetic sweeteners	1
	5	Safety of specified pesticides and materials used in place of pesticides	1
	5	Polycyclic aromatic hydrocarbons	1
	5	Synergy effect	1
42	4	Food poisoning due to hepatitis E virus	1
	4	Genetically modified food crops	2
	4	Methylmercury (fetus is a high-risk group)	1
	4	Health effects of residual veterinary drugs	1
	4	Carcinogenicity of red meat and processed meat	1
47	3	Trans fatty acids in foods (novel foods)	2
	3	Meat safety during avian influenza, foot-and-mouth disease, etc.	1

**Table 2:** c. (*Continued*)

Round 2			Round 1
Rank	Scores	Topics	Number of people on the same topic
49	2	Food poisoning by *Anisakidae*	1
	2	Artificial coloring materials	1
	2	Energy and vitamin drinks	1
	2	Hygiene management of eat-in equipment	1
	2	Health effects due to overeating (overdose) and uneven diet	2
54	1	Food management	1
	1	Drinking spring water, river water, etc.	1
	1	Problems in Toyosu Market	1
57	0	Food poisoning by natural toxins (animal based)	1
	0	Food poisoning by parasites	1
	0	Safety of pickles (lightly salted pickles, etc.)	1

**Table 3:** a. Round 1 and Round 2 topics ranked by the LGO group

Round 2^a)^			Round 1^a)^
Rank	Scores^b)^	Topics	Number of people on the same topic^c)^
1	69	Risks of eating raw meat	5
2	67	Food poisoning by *C. jejuni* and *C. coli*	15
3	60	Risk analysis	2
4	59	The concept of food safety and food safety	1
5	50	The concept of food safety	2
6	36	Prevention and measures for food poisoning	5
7	35	Food poisoning by *Norovirus*	13
	35	Health foods	8
9	31	Institutionalization of hazard analysis and critical control point (HACCP)	6
10	26	Food label	7
11	24	Food allergies	2
12	21	Food additives	10
13	20	Food poisoning by enterohaemorrhagic *E. coli*	1
14	19	Information literacy	1
15	14	Imported food safety	5
16	13	Risks of eating raw wild animal meat	3
17	12	Types of food poisoning and the conditions under which each occurs in foods	1
18	10	Pesticide residue	2
19	9	Radioactive materials in foods	2
20	8	Genetically modified foods	3
21	6	Food poisoning by natural toxins (animal- and plant-based)	1
22	3	Inspection of radioactive materials	1
23	2	Food poisoning by *Anisakidae*	6
24	1	Changes in causes due to the time of occurrence of food poisoning	1
25	0	Food poisoning by *Kudoa septempunctata*	1
	0	Food poisoning by natural toxins (plant-based)	3
	0	Mycotoxin	1
	0	Acrylamide	1

^a)^ Number of participants in Round 1 and Round 2 were 26 and 23, respectively.

^b)^ There were 15 consenters on the first topic, and no consenters on the 25th topic.

^c)^ The total number of consenters for all topics was 109.

The EC group listed 59 topics in Round 1 (Tables 2a–2c). The most frequently chosen topic (7 participants) was “food poisoning caused by natural toxins (plant-based)”. LGO group listed a total of 28 topics (Table 3). The topics “food poisoning by *Campylobacter jejuni* and *Campylobacter coli*”, “food poisoning caused by *Norovirus*”, and “food additives” were chosen by more than 10 participants. FSM group listed 48 topics (Tables 4a–4b). The most frequently chosen topic (9 participants) was “food poisoning caused by *Norovirus*”. Since some of the topics in Round 1 were unscored, the numbers of topics scored in Round 2 were 56, 24, and 39 in the EC, LGO, and FSM groups, respectively. In the EC group, “concept of risk” had the highest score of 70. In the LGO group, “risks of eating raw meat” had the highest score of 69. In the FSM group, “food safety and food relief” showed the highest score of 58. When compared the total number across the board of groups, the highest number identified in Round 1 was below of the highest score in Round 2.

**Table 4:** a. Round 1 and Round 2 topics ranked by the FSM group

Round 2^a)^			Round 1^a)^
Rank	Scores^b)^	Topics	Number of people on the same topic^c)^
1	58	Food safety and food relief	1
2	55	Food poisoning by enterohaemorrhagic *E. coli*	3
3	47	Food poisoning by *Norovirus*	9
4	33	Food poisoning by eating raw meat	2
5	27	Food poisoning by microorganisms	1
	27	Public awareness activities	1
7	26	Food poisoning by *C. jejuni* and *C. coli*	3
8	23	Imported food safety	2
9	21	Health foods	6
	21	Correct knowledge of food labeling	6
11	20	Institutionalization of HACCP	2
12	19	Infant botulism by honey	1
13	17	Radioactive materials in foods	4
14	16	Food additives	5
15	15	Food poisoning by *C. perfringens*	1
16	14	Food allergies	4
	14	Pesticide residue	2
18	13	Food defense	1
19	11	Food poisoning by *Anisakidae*	3
	11	Food poisoning by natural toxins (plant-based)	3
	11	Secondary infection of food poisoning	1
	11	Health management of food manufacturing employees	1
23	9	Mycotoxin	2
	9	Food poisoning by histamine	2
	9	Antimicrobial resistant bacteria	1
	9	Best-before date	1
	9	Hygiene management in food manufacturing and distribution	2

^a)^ Number of participants in Round 1 and Round 2 were 24 and 23, respectively.

^b)^ There were 15 consenters on the first topic, and no consenters on the 40th topic.

^c)^ The total number of consenters for all topics was 101.

**Table 4:** b. (*Continued*)

Round 2			Round 1
Rank	Scores	Topics	Number of people on the same topic
28	8	Food poisoning by *S. aureus*	1
	8	Management of food storage	4
30	7	Food poisoning by *B. cereus*	1
	7	Bottled mineral water	1
32	6	Genetically modified crops	1
	6	Avian influenza	2
	6	Large intake of olive oil	1
35	5	Rot and fermentation	2
36	3	Toxin-type food poisoning	1
37	2	Fish parasite	4
	2	Acrylamide	1
39	1	Food poisoning by *Salmonella*	2
40	0	Food poisoning by *Listeria*	2
	0	Food poisoning by natural toxins	1
	0	Puffer poison	1
	0	Food additive standards	1
	0	Use of drugs in imported chicken	1
	0	Standards ensuring the safety of food equipment, containers, and packages	1
	0	Gluten-free food	1
	0	Liquid infant milk	1
	0	Differentiating beverage types for early symptoms of dehydration	1

The number of topics scored in Round 3 was 36, 24, and 35 in the EC, LGO, and FSM groups, respectively. All the topics in the LGO group were scored. Topics with scores of 10 or more in Round 3 are shown in Tables 5–7 with the scores and percentages of the total. Topics with scores up to 10 accounted for 87.0%, 93.2%, and 86.0% of the total scores of the scored topics in the EC, LGO, and FSM groups, respectively.

**Table 5.** Round 3 rankings by the EC group (topics that scored 10 or more among the 36 topics scored in Round 3)

Round 3^a)^				Round 2
Rank	Scores^b)^	%	Topics	Scores
1	88	13.8	Concept of risk	70
	88	13.8	Health foods	55
3	49	7.7	Safety costs and relevant risk management	31
4	43	6.8	Difference between safety and relief	53
5	36	5.7	Food poisoning by natural toxins (animal- and plant-based)	29
6	31	4.9	Food additives	21
	31	4.9	Food allergies	19
8	28	4.4	Causes and prevention of food poisoning	19
9	27	4.2	Genetically modified foods	19
10	19	3.0	Difference between acute and chronic effects	19
11	18	2.8	Imported food safety	19
12	17	2.7	Correct knowledge of food labeling	16
13	16	2.5	Food poisoning by microorganisms	18
14	14	2.2	Artificial products vs. natural products	27
	14	2.2	Radioactive materials in foods	23
	14	2.2	Risk of sweetened beverages	13
17	11	1.7	Eating raw food (meat/fish)	19
18	10	1.6	Prioritizing concept in food safety standards and risk communication	15
		87.0	Total	

^a)^ Number of participants in Round 3 was 24.

^b)^ The scores of the omitted topics (19th [2 topics] to 36th) were from 9 to 1, respectively. The scores of the other 20 topics were all zero.

“Concept of risk” was included in the top five topics in all groups. Specifically, the EC group had “concept of risk” (1^st ^place) and “difference between safety and relief” (known as ‘Anzen and Anshin’ in Japanese) (4^th^ place) (Table 5), the LGO group had “concept of safety and relief” (4^th^ place) (Table 6), and the FSM group had “safety and relief” (1^st^ place) (Table 7). The topics related to food poisoning accounted for three of the top five topics in the LGO group. These specific topics were “risks of eating raw meat”, “food poisoning by *C. jejuni* and *C. coli*”, and “food poisoning caused by *Norovirus*” (Table 6). In the EC and FSM groups, “health foods” was ranked 1^st^ and 4^th^ places, respectively (Tables 5–7). EC group had “radioactive materials in food” at 14^th^ ranking (Table 5). FSM and LGO groups, this topic was ranked below 10^th^ place.

**Table 6.** Round 3 rankings by the LGO group (topics scored 10 or more among the 24 topics scored in Round 3)

Round 3^a)^				Round 2
Rank	Scores^b)^	%	Topics	Scores
1	112	15.1	Risks of eating raw meat	69
2	80	10.8	Food poisoning by *C. jejuni* and *C. coli*	67
3	78	10.5	Prevention and measures for food poisoning	36
4	67	9.1	Concept of safety and relief	59
	67	9.1	Food poisoning by *Norovirus*	35
6	57	7.7	Concept of food safety	50
7	43	5.8	Risk analysis	60
8	39	5.3	Food poisoning by enterohaemorrhagic *E. coli*	20
9	32	4.3	Health foods	35
10	27	3.6	Institutionalization of HACCP	31
	27	3.6	Food labeling	26
12	20	2.7	Information literacy	19
13	16	2.2	Food additives	21
14	14	1.9	Food allergies	24
15	11	1.5	Imported food safety	14
		93.2	Total	

^a)^ Number of participants in Round 3 was 27.

^b)^ The scores of the omitted topics (16th to 23rd [2 topics]) were from 8 to 3, respectively. The scores of the other 3 topics were all zero.

**Table 7.** Round 3 rankings by the FSM group (topics scored 10 or more among the 35 topics scored in Round 3)

Round 3^a)^				Round 2
Rank	Scores^b)^	%	Topics	Scores
1	84	12.6	Safety and relief	58
2	68	10.2	Food poisoning by enterohaemorrhagic *E. coli*	55
3	53	8.0	Food poisoning by *Norovirus*	47
4	46	6.9	Health foods	21
5	43	6.5	Correct knowledge of food labeling	21
6	36	5.4	Imported food safety	23
7	29	4.4	Public awareness activities	27
8	27	4.1	Hygiene management in food manufacturing and distribution	9
9	25	3.8	Institutionalization of HACCP	20
10	24	3.6	Food allergies	14
11	20	3.0	Secondary infection of food poisoning	11
12	18	2.7	Food defense	13
13	17	2.6	Food poisoning by microorganisms	27
	17	2.6	Rot and fermentation	5
15	15	2.3	Food additives	16
16	14	2.1	Food poisoning by *C. jejuni* and *C. coli*	26
17	12	1.8	Actual conditions and countermeasures of food-resistant drug-resistant bacteria	9
18	11	1.7	Food poisoning by eating raw meat	33
	11	1.7	Infant botulism by honey	19
		86.0	Total	

^a)^ Number of participants in Round 3 was 24.

^b)^ The scores of the omitted topics (20th [3 topics] to 35th) were from 9 to 1, respectively. The scores of the other 4 topics were all zero.

**Table 8.** Top five topics in Round 3 and the reasons provided in Round 1 for each group^8^^)^

Expert Committees			Local Government Officials			Food Safety Monitors			
Rank	Topics	Reasons	Rank	Topics	Reasons	Rank	Topics	Reasons	
1	Concept of risk	The concept of risk has not yet permeated throughout whole society. Many people seek zero risk.	1	Risks of eating raw meat	Consumers and businesses do not have enough knowledge about the risks of eating raw meat; many consumers have a misunderstanding of the risks.	1	Safety and relief	There is confusion about the science-based “safety” factor and the psychological factor “relief”; there are excessive demands for foods.	
1	Health foods	There are few opportunities to explain the scientific data to the general public. Many people have the mistaken notion that there are no side effects with excessive intake.	2	Food poisoning by *C. jejuni* and *C. coli*	Consumers and businesses do not have a good understanding of the risks of eating raw meat. There is a divergence between the government and consumers.	2	Food poisoning by enterohaemorrhagic *E. coli*	No risk is known (for enterohemorrhagic *E. coli*). Not familiar.	
3	Safety costs and relevant risk management	It is necessary to explain that unlimited costs would be incurred if too much safety was pursued, that relevant risk management can ensure efficient safety.	3	Prevention and measures for food poisoning	The measures and information on food poisoning are not provided sufficiently to consumers and businesses. It is necessary to publicize the importance of preventive measures for food poisoning.	3	Food poisoning by *Norovirus*	It is important to formulate countermeasures for each food manufacturing process. There is no education provided for food manufacturers employees.	
4	Difference between safety and relief	The general public seems to be confused because they cannot distinguish between safety and security. They need correct explanations.	4	Concept of safety and relief	It is necessary to provide the correct information on the mechanisms used for risk assessment and standard setting.	4	Health foods	There is a lack of consumer education on health foods which may have a potential adverse effect on health.	
5	Food poisoning by natural toxins (animal-and plant-based)	Not enough information is available to consumers.	4	Food poisoning by *Norovirus*	Information has not been widespread due to insufficient understanding by consumers and businesses. There are no decisive prevention measures for food poisoning.	5	Correct knowledge of food labeling	Allergy labels are inconsistent and difficult to understand. It is necessary to evaluate the degree of consumer understanding of food functional labeling.	

Table 8 shows the top five topics in Round 3, with reasons of their choice by each group in Round 1^7^^)^.

## Discussion

The use of the Delphi method can minimize the unnecessary influences such as the biases based on individual interest in each topic or common sense. The European Food Safety Authority (EFSA) presented an agenda that read “priority activities and initiatives that are likely to have the greatest impact on strengthening risk assessment and risk monitoring”, after conducting a survey among experts by utilizing the Delphi method to risk assessment in 2016^8^^)^. Following the idea of EFSA, the efficacy and effectiveness of the Delphi method was evaluated by the FSCJ survey. The participants in the FSCJ survey included many experts in the food safety and hygiene in all the three groups, i.e., EC, LGO, and FSM. The participants came from various backgrounds and occupations, ranging from medicine, to veterinary medicine, pharmacy, and agriculture.

Furthermore, in contrast to group interviews, the present study used website system to allow low-cost collection of replies from broad geographical locations. This survey achieved high response rates of 79–100% (Table 1), which is a supporting condition to gain reliable outcome.

One of the characteristics of this survey’s results is that the primary appearance of topics is independent of specific risks or hazards for the top five items within each group (Tables 4–6). The top ranking of the EC group, “concept of risk”, might have a link with topic of “the concept of risk has not yet permeated throughout whole society, and many people seek risk-zero” (Table 8). This “concept of risk” is expressed as the probability and the extent of influences on human health caused by the presence of hazards in foods. Experts also found it difficult to recognize these risks as the consumer’s own problem. Masuyama et al^4^^)^ reported that the “concept of risk” was ranked the first, which indicates a gap between the consumers and experts. This gap has not yet been resolved.

FSM group had “safety and relief” at top rank, while the EC group had “difference between safety and relief” at 4^th^ rank. The topic “concept of safety and relief” was ranked 4^th^ among the LGO group. All the groups shared the similar reason for these choices, such as requesting the government to provide clearer information to consumers as they are confused with safety and relief (Table 8). Another reason was “an excessive risk-zero was required for relief”. The EC group’s results indicated that this confusion is attributable to the media^4^^)^, as the merits and demerits of the media ranked at 5^th^. The LGO and FMS groups, however, did not describe it as the media. We considered that the FSCJ should communicate directly to resolve the consumer’s misunderstandings without relying on the media.

“Safety costs and relevant risk management” was ranked 3^rd^ in the EC group (Table 5). This is the first time that this topic was ranked on the list in this survey. The EC group’s opinion was that the persuasion of too much safety requires unlimited costs, thus relevant risk management to balance the cost would yield efficient safety (Table 8). We considered that the cost-effectiveness of food safety should be further analyzed, and the results need to be communicated to consumers.

Specific hazards were also notable topic to in Round 3. The topic “hazards related to food poisoning caused by microorganisms” was ranked 5^th^ by the EC group, 1^st^ to 3^rd^ and 5^th^ by the LGO group (Table 6), and 2^nd^ and 3^rd^ by the FSM group (Table 7). These were practical hazards related to the “risks of eating raw meat” and “food poisoning by *C. jejuni* and *C. coli*. Both the LGO and FSM groups argued that consumers and food business operators had limited chances to receive accurate information about the hazards of “eating raw meat” and “under-heated meat” (Table 8). The LGO group participants were mainly daily operators of food poisoning incident tasks and provision providers of monitoring and guidance to restaurants, so in a way characterized as those who have jobs related the topics. “Food poisoning” was ranked 1^st^ by the FSM group in annual surveys concerning hazards^2^^)^. It is reasonably presumed that they had practical work experiences on food poisoning isses. It is thus reasonable that both groups raised topics providing information to consumers and businesses from a practical perspective on how to prevent food poisoning. Food poisoning by enterohemorrhagic *Escherichia coli* occurred before Round 2 questioner took place^9^^)^. The incident caused serious illness in several patients and a young child died. The process of incident was covered repeatedly by the media before the Round 3 questionair took place. This might also have impacted on the outcome of our survey results. According to a report from the Ministry of Health, Labour and Welfare (MHLW)^10^^)^, approximately 1,000 cases of food poisoning occur annually, and approximately 20,000 patients are affected each year. In a survey by Nakagaki et al^3^^)^, the 1^st^ and 2^nd^ rankings were those related to food poisoning. The MHLW, the FSCJ, and the National Consumer Affairs Center of Japan (NCACJ) have been issuing warnings about food poisoning. The FSCJ has up-dated the risk profiles of *C. jejuni*^11^^)^ and *Norovirus*^12^^)^. There were, however, no noticeable changes in number of cases or patients of these types of food poisoning. The government needs to continue communication about the risks of food poisoning and mitigation measures.

The topic “health foods” ranked 1^st^ among the EC group (Table 5) and 4^th^ among the FSM group (Table 7). Both groups were concerned about the health hazards of consumers’ overdosing “health foods” (Table 8). The EC group stated that they had few opportunities to give scientific data to consumers. The FSM group stated that consumers were unfamiliar with the labeling systems of “health foods” and lacked the knowledge to identify inferior goods. In the annual FSM survey^2^^)^, “health foods” ranked at 2^nd^ in years from 2015 through 2017. Cases of adverse effect on health linked to “health foods” have been reported by the MHLW, and the FSCJ^13^^)^ issued a warning regarding those matters to the public in 2015. After Round 1 questioner, the NCACJ issued a warning to the public about the potential adverse effect on health when consumed *Pueraria mirifica*^14^^)^. The adverse effect on health was also covered by media, so this coverage might have influenced on the outcome of Round 3. Regarding “health foods”, we should provide information including up-dated scientific information, as well as calling on caution to overdose, and disseminating information about labeling system.

The scores for “radioactive materials” were less than 10 among the FSM and LGO groups. FSM annual survey^2^^)^ ranked pollutants and pesticide residues as hazards in the 2000s and radioactive substances in 2011. In this survey, however, the hazards of these materials were low in the Round 2. Abe et al^15^^)^ reported that the pollutant status of these material as hazards and their corresponding risks have gradually gained understanding, also indicated by the steady decrease in anxiety scores from 2004 to 2018. Regarding material hazards, we consider that governments should continue to disseminate information on exposure states and risk assessments.

In this survey, conceptual topics related to food risks were ranked at the top of all groups. These topics had also appeared in previous surveys among professionals, but the problems have not been improved. The annual survey of FSMs on various issues in daily life^2^^)^ showed that concerns about the levels of food safety were lower than that of natural disasters and environmental problems. These results indicated that consumers were not informed about food safety-related risks properly, possibly because of only few opportunities to learn about the concept of food safety during pre-adulthood in Japan. The curriculum guidelines of high school home economics^16^^)^ states that the upper limits of standard intake are set on nutrients of only “special purpose foods”, “health functional foods”, and “supplements”. The curriculum guidelines of Health and Physical Education^17^^)^ state that standards have been set to ensure food safety and that food hygiene protocols are being carried out. However, consumers need to understand that, even if food poses a risk of a hazard, they should act according to the degree of risk based on their intake.

The FSCJ, in November 2018, concluded that the priority themes of risk communication to be “risk analysis” and “basic concept of food safety”^18^^)^. This conclusion was reached based on the further needs of consumers to understand food safety, which at that moment was not always sufficient. The experts concluded that it was an important topic from the survey by the Delphi method. At the same time, food poisoning caused by *Campylobacter* was also a priority theme. We consider that the method of communication on information to consumers needs to be adequately concrete. We would consider that each scientific group can offer further contribution, such as the EC experts’ providing scientific evidence, the LGO group’s offering management guidance for businesses, and the FSM group’s having daily information penetrated to the public including families and workplaces. Readiness of materials and creating more communication channels by the FSCJ would future prompt the influencers in each group to spread the messages effectively. Direct dissemination by the FSCJ to the consumers is also required, in order to increase the frequency of spreading easy-to-understand information through various media.

## Conclusion

In this study with the Delphi method, we identified the prioritized order of information to be communicated with consumers, based on the expert opinions. The top priorities included “risk concept” and “difference between safety and relief”, and the top hazards were “food poisoning” and “health foods”. The FSCJ, with these pieces of information, will be enabled to focus on identified topics to disseminate to the general public.
